# Observation of a hybrid state of Tamm plasmons and microcavity exciton polaritons

**DOI:** 10.1038/srep34392

**Published:** 2016-10-04

**Authors:** SK. Shaid-Ur Rahman, Thorsten Klein, Sebastian Klembt, Jürgen Gutowski, Detlef Hommel, Kathrin Sebald

**Affiliations:** 1 Semiconductor Optics, Institute of Solid State Physics, University of Bremen, Bremen, 28334, Germany; 2 Semiconductor Epitaxy, Institute of Solid State Physics, University of Bremen, Bremen, 28334, Germany

## Abstract

We present evidence for the existence of a hybrid state of Tamm plasmons and microcavity exciton polaritons in a II-VI material based microcavity sample covered with an Ag metal layer. The bare cavity mode shows a characteristic anticrossing with the Tamm-plasmon mode, when microreflectivity measurements are performed for different detunings between the Tamm plasmon and the cavity mode. When the Tamm-plasmon mode is in resonance with the cavity polariton four hybrid eigenstates are observed due to the coupling of the cavity-photon mode, the Tamm-plasmon mode, and the heavy- and light-hole excitons. If the bare Tamm-plasmon mode is tuned, these resonances will exhibit three anticrossings. Experimental results are in good agreement with calculations based on the transfer matrix method as well as on the coupled-oscillators model. The lowest hybrid eigenstate is observed to be red shifted by about 13 meV with respect to the lower cavity polariton state when the Tamm plasmon is resonantly coupled with the cavity polariton. This spectral shift which is caused by the metal layer can be used to create a trapping potential channel for the polaritons. Such channels can guide the polariton propagation similar to one-dimensional polariton wires.

Exciton polaritons are half light, half matter bosonic quasiparticles, resulting from the strong light-matter coupling between the quantum well (QW) excitons and the cavity photons in a microcavity (MC). The effective mass of microcavity exciton polaritons is exceedingly small, typically in the order of 10^−4^ times the bare electron mass. This fascinating property allows for exciton polaritons to undergo a condensation at temperatures ranging from tens of Kelvin^1^,^2^ up to 300 K^3^,^4^,^5^,^6^,^7^. Moreover, a number of interesting phenomena have been observed in such MC structures like parametric amplification^8^, superfluidity^9^, and polarization bistability^10^.

In order to realize polariton-based devices operating at room temperature large exciton binding energies and high oscillator strengths are of essential importance. In this context ZnSe-based MCs are of particular interest since they possess these fundamental properties. For this material system a promising Rabi splitting energy of about 19 meV for a MC with 3 QWs^11^ and polariton lasing^12^,^13^ have already been reported.

One crucial challenge is to control and manipulate the polariton eigenstate what is extremely important for fundamental physics as well as for the realization of polariton-based devices in future. Owing to the hybrid nature of exciton polaritons, spatial confinement can be achieved via their excitonic as well as their photonic component^14^. A variety of techniques for polariton trapping has been proposed and experimentally demonstrated. These concepts are based on local strain^2^, surface acoustic waves^15^, an exciton reservoir^16^, photonic disorder traps^17^, photonic crystals^18^, cavity thickness modulation^19^, and pillar cavities^20^. However, for practical applications of polariton-based devices a simple design would be advantageous. One approach is the deposition of metal strips, resulting in a local energy blueshift of the cavity mode and thus the lower polariton. In such a way potential barriers of about 200 *μ*eV^21^ can be created. This trapping potential is rather small for polaritonic quantum devices. However, by employing Tamm plasmons (TPs)^22^,^23^ which are formed at the interface between a metal and a distributed Bragg reflector (DBR) the trapping potential can be enhanced. The utilization of these TP resonances in order to observe strong coupling with QW excitons has already been reported^24^,^25^,^26^,^27^. An interesting technique has been proposed theoretically by Kaliteevski *et al*.^28^ by employing a hybrid state of a TP and a microcavity exciton polariton where the TP mode is resonantly coupled with the exciton-polariton mode. For this concept it was shown that the spectral modulation of the lowest polariton energy determined by the interaction with the metal layer is much larger compared to the non-resonant modulation of the cavity polariton^21^.

In this contribution, we report on the experimental observation of a hybrid TP-microcavity exciton polariton state in a ZnSe-based MC. This structure will exhibit a sufficiently large lateral confinement potential for the lowest cavity polariton state if a metal layer is used. Such confinement potential is important for creating one-dimensional channels to control the flow of polaritons in a defined direction.

## Results

**Figure 1:**
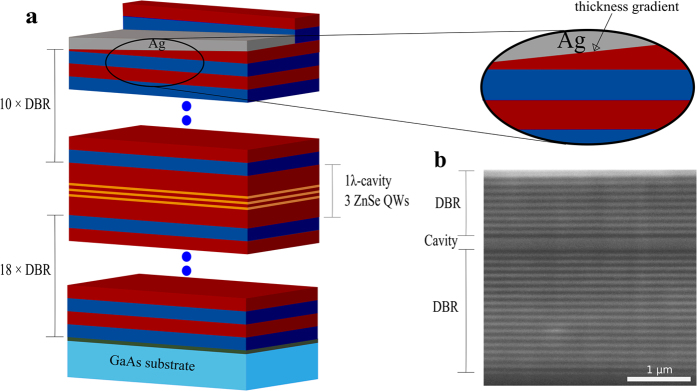
(**a**) Schematic of the investigated MC structure. The sample possesses a thickness gradient for the top DBR layer and it is covered by a 40 nm of Ag layer. (**b**) Scanning electron microscope image of the as-grown MC structure.

**Figure 2:**
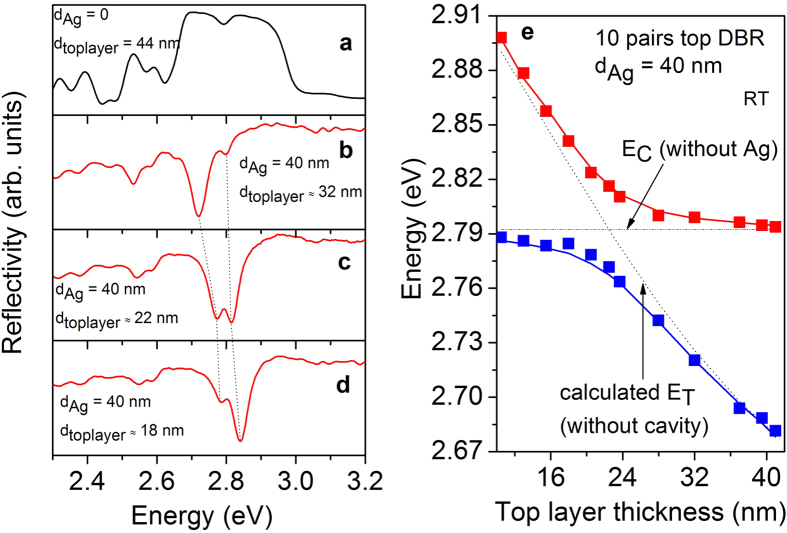
Measured microreflectivity spectra of the MC sample at RT (**a**) without Ag, (**b**) with a 40 nm Ag. The thickness of the top DBR layer is approximately 32 nm. Both the TP and the cavity resonances are observed at the low energy side of the photonic stopband, (**c**,**d**) microreflectivity spectra measured at RT for different top layer thicknesses, resonances shift to higher energies. (**e**) Measured (squares) and calculated spectral positons (solid lines) of the resonances of the Ag covered MC sample for different thicknesses of the top DBR layer. The calculated bare TP and the bare cavity modes are shown as dotted black and red line, respectively.

**Figure 3:**
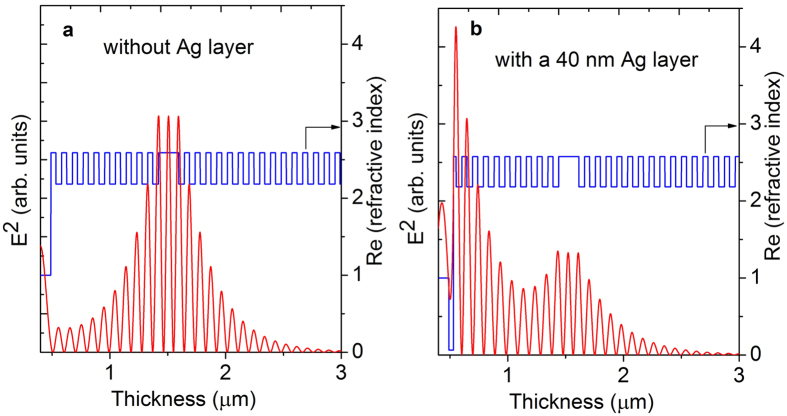
Calculated profiles of the squared electric field distribution and the refractive index for (a) the cavity photon mode localized between two DBRs at an energy of 2.7924 eV, (b) the lowest eigenstate coupled mode of the TP and the cavity mode with an energy of 2.768 eV. The TP mode is tuned into resonance with the cavity mode by adjusting the thickness of the top DBR layer.

Figure 1(a) represents the schematic of the MC sample with 40 nm Ag layer. The as-grown MC sample structure consists of an 11-fold top DBR, a 1*λ* cavity layer and an 18-fold bottom DBR. In Fig. 1(b), a cross-section scanning electron microscope (SEM) image of the MC structure is depicted. The number of layer pairs for the top DBR was reduced to achieve a 10-fold DBR by an etching process accompanied by the creation of a thickness gradient of the top layer of the DBR as shown in Fig. 1(a) in order to tune the eigenenergy of the TP resonance (see methods). Figure 2(a) shows the microreflectivity spectrum of the uncovered MC sample measured at room temperature. A photonic stop-band width of approximately 310 meV is observed and the cavity resonance can be identified at 2.792 eV. At room temperature the QW emission energy is largely detuned in the order of 80 meV relative to the cavity resonance and the linewidth is 36 meV (measured for a reference sample). Hence, the bare interaction between the cavity resonance and the TP mode can be investigated. However, at room temperature the direct band-to-band transitions can lead to additional absorption which can be safely neglected for the following low temperature investigations. Figure 2(b) represents the microreflectivity spectrum of the Ag covered MC sample, where the metal adjacent semiconductor layer thickness was reduced to approximately 32 nm by chemically assisted ion beam etching (CAIBE), starting from an initial thickness of 44 nm. In this spectrum two resonances are observed at the low-energy side of the stop-band. One resonance minimum at 2.72 eV shows a pronounced transmission. The origin of this resonance is due to the formation of the TP mode at the interface between the metal and the DBR. A second resonance minimum is observed at 2.798 eV which is nearly at the spectral position of the bare cavity mode. Both resonances shift to higher energies when the top DBR layer thickness is further reduced (shown in Fig. 2(c,d)). However, the spectral shift of the resonances is different. The shift of the first resonance (TP mode) depends on the thickness of the metal-adjacent semiconductor layer^22^. The shift of the second resonance (cavity mode) is caused by the coupling to the TP mode. Evidence of this coupling is given in Fig. 2(c) where two nearly symmetric resonances are observed for a top DBR layer thickness of approximately 22 nm. The dependancy of these two reflectivity minima on the top DBR layer thickness is shown in Fig. 2(e) in comparison to the calculated spectral positions of the resonances based on the transfer matrix method. A clear anticrossing is observed between the two modes in excellent agreement with the calculation. The splitting energy between the modes amounts to about 44 meV and is deduced from the measurement when the TP and cavity modes are in resonance. Our findings are comparable to those on a sample for which the metal layer was placed in between the cavity and the DBR^29^. Until now we have discussed our results for the MC sample with a 10-fold top DBR as shown in Fig. 1(a). Additionally, calculations reveal that the splitting energy can be varied by changing the number of the top DBR pairs. This is confirmed by measuring a sample with an 11-fold top DBR (see supplementary information) yielding a splitting energy of about 34 meV. Hence, the splitting energy decreases with increasing the number of top DBR pairs. Figure 3 depicts the calculated squared electric field distribution in one and the same MC structure but without and with Ag layer. As expected, the field maximum can be observed at the center of the cavity layer in the absence of the Ag layer (Fig. 3(a)). However, after introduction of the Ag layer the hybrid mode exhibits two field maxima. One is located at the metal-DBR interface and the other at the center of the cavity (Fig. 3(b)). The electric field is confined at the interface between the metal and the DBR what is a clear evidence for the existence of the TP mode in our structure. One of the main advantages of the hybrid TP-cavity compared to the bare TP system is the reduction of absorption in the metal film, and consequently, an enhancement of the Q factor^30^. In the case the TP and cavity mode are in resonance (as shown in Fig. 2(c)), the two hybrid modes are nearly symmetric and thus possess comparable Q factors. However, the Q factor of one of these modes is increased by a factor of 2 with respect to the bare TP mode (a comparison of simulated reflectivity spectra is shown in the supplementary information). Such an increase of the Q factor implies less absorption losses for this hybrid system.

**Figure 4:**
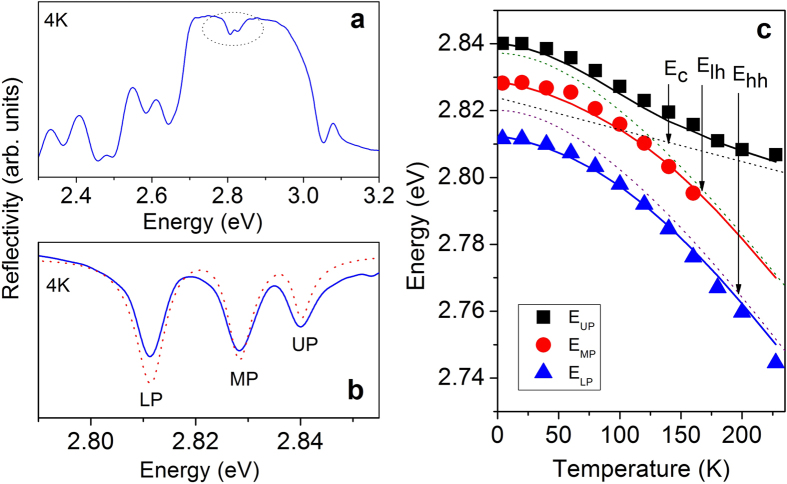
(**a**) Microreflectivity spectrum of the MC without Ag layer measured (solid line) at T = 4 K. (**b**) Spectrally highly resolved microreflectivity in the vicinity of the exciton-photon resonance, compared with the calculated spectrum (dotted line), (**c**) Energies of the upper (black), middle (red), and lower polariton (blue-symbols) reflectivity resonances as function of temperature in comparison with the calculation (solid lines). Uncoupled *X*_*hh*_ (*X*_*lh*_) as purple (olive) dotted line and calculated cavity mode (black-dotted line) as a function of temperature are shown as well.

The interactions between the QW excitons and cavity mode have been investigated at low temperatures since the resonances are largely detuned relative to each other at room temperature in our MC sample. Figure 4(a) represents the overview spectrum (spectral resolution SR = 11 meV) of the MC sample without Ag at T = 4 K, where the cavity mode and the QW heavy-hole excitons are expected to be nearly in resonance. In addition, the region of particular interest within the reflectivity spectrum is shown spectrally highly resolved (SR = 0.1 meV) in Fig. 4(b). Three reflectivity minima can be identified at the spectral positions 2.8105 eV, 2.8283 eV, and 2.8403 eV, which are attributed to the lower, middle, and upper polariton, respectively. The observed reflectivity spectrum can be reproduced by a transfer matrix calculation (dotted line). In the calculation oscillator strengths of f = 1.78^13^ cm^−2^ (ref. 31) for the heavy-hole exciton (*X*_*hh*_) and f/3 for the light-hole exciton (*X*_*lh*_) are implemented as well as the QW excitonic emission linewidth (FWHM = 3 meV for both *X*_*hh*_ and *X*_*lh*_) which is taken from the reference sample. In Fig. 4(c) the evolution of these three resonances as function of the temperature is displayed. For comparison, the emission energies of *X*_*hh*_ and *X*_*lh*_ for these temperatures taken from a reference sample (reflectivity measurement) are plotted as well (dotted lines). Moreover, the calculated spectral shift of the bare cavity mode is shown as a dashed line. In the calculation, the temperature dependent refractive index change (dn/dT) is assumed to be constant^32^. Two anticrossings of the cavity mode with *X*_*hh*_ and *X*_*lh*_ are observed. The experimental findings are in good agreement with the calculation based on the three-coupled-oscillators model (solid line). Splitting energies of *ħ*Ω_*hh*_ ≈ 17.5 meV and *ħ*Ω_*lh*_ ≈ 12 meV are derived from the measurement at 20 K, where the cavity mode and *X*_*hh*_ possess a small detuning of ~2.5 meV.

**Figure 5:**
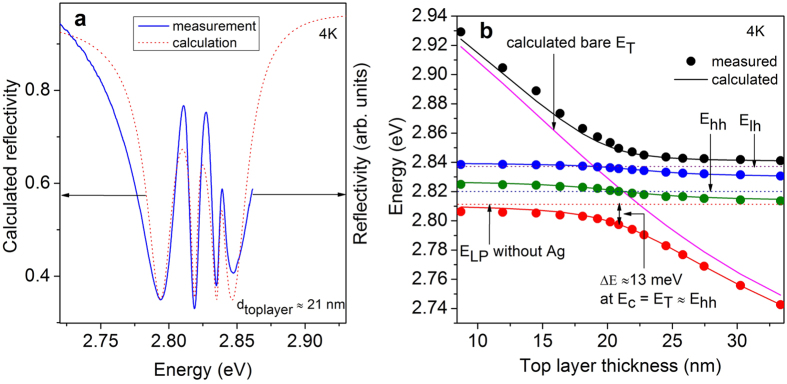
(**a**) Region of interest of the measured microreflectivity spectrum (solid line) of the Ag covered MC sample at 4 K in comparison to the calculated spectrum (dotted line). (**b**) The spectral position of the resonances (dots) as a function of the tuning of the TP mode (by choosing different top layer thicknesses), compared with the calculation (solid line). The calculated bare TP mode (solid magenta line) and the lower polariton energy position (dotted line) of the cavity polaritons are plotted as well.

Up to now, we have shown evidence that a strong coupling regime exists in this sample at low temperatures. As next step, we investigate the influence of TPs on the eigenstates of the exciton polaritons. In Fig. 5(a) the microreflectivity spectrum of the MC covered with Ag is depicted as measured at 4 K, where the top layer thickness of the DBR is approximately 21 nm thick. Four resonances can be observed in the reflectivity spectrum which is comparable to the calculated spectrum. The difference between the measurement and the calculation, especially the spectral linewidth of the lower and upper resonances, is mainly due to metal oxidation^24^ and absorption losses which were not taken into account for the calculation. The presence of the TP mode which forms a hybrid TP-microcavity exciton polariton state is identified as being the reason for the formation of four resonances. This is mathematically comparable to the situation of two coupled cavities^33^, although the sample setup is quite different. The properties of these hybrid states can be investigated by tuning the eigenstate of the bare TP mode. Figure 5(b) shows the hybrid modes as a function of the thickness of the top DBR layer adjacent to the metal. The solid magenta line represents the shift of the calculated bare TP eigenenergy in dependence on the top layer thickness. The measured eigenenergies of the hybrid system are compared to the calculated ones by solving the four-coupled-oscillators equation,


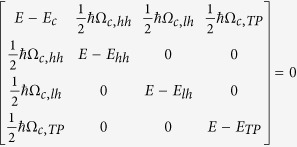


where *E*_*c*_, *E*_*hh*_, *E*_*lh*_, and *E*_*TP*_ are the eigenenergies of the bare cavity mode, of *X*_*hh*_, *X*_*lh*_, and of the TP mode, respectively. 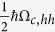, 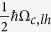, and 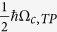 are the coupling strengths of the cavity photons to *X*_*hh*_, *X*_*lh*_, and to the TP mode, and correspond to half the splitting energy. The measured values of 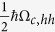 = 17.5/2 meV, 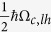 = 12/2 meV, and 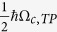 = 44/2 meV are employed in the calculation. The experimental observation of three anticrossings is nicely reproduced by the coupled-oscillators-model calculations (see Fig. 5(b)). Further, this multiple anticrossing feature agrees with a previous theoretical report^28^. If the exciton (*X*_*hh*_), cavity, and TP mode are nearly in resonance (*E*_*hh*_ ≈ *E*_*C*_ = *E*_*T*_ at the top layer thickness of approximately 21 nm), the lowest hybrid mode will be red shifted by ΔE ≈ 13 meV with respect to the lower cavity polariton state without Ag (indicated by the horizontal dotted line). The corresponding value of ΔE = 12.9 meV deduced from the simulation based on the transfer matrix method is in good agreement with the experimental finding as well as with the result obtained using the coupled-oscillators model. This offset ΔE can be utilized as a confinement potential for the cavity polariton when structuring just the metal part of the hybrid metal-MC structure in terms of gratings or disks. It should be noted that a reduction of the lateral size of the metal layer can also yield a confinement of the photonic component as it has been shown for the bare TP system^34^. As a result, the overall confinement for this hybrid system can be further enhanced when using structured metal layers.

When the TP mode is strongly detuned from the cavity polariton mode the effect of the metal is a blue shift of the lower polariton mode. However, if the TP mode is in resonance with the cavity polariton mode, the lowest hybrid mode will be red shifted with respect to the bare polariton mode. The approach of such resonant modulation of the lower polariton mode provides a larger in-plane confinement potential for the polaritons. The potential depth ΔE depends on the exciton oscillator strength and on the number of top DBR pairs. The calculated Q factor for the investigated MC structure is about 1000. One should consider the fact that the cavity Q factor increases with the number of top DBR pairs but, at the same time, the depth of the confinement ΔE decreases. According to the calculation, a confinement depth of ΔE = 3 meV can be obtained by utilizing a 16-fold top DBR where the cavity Q factor increases by a factor of 3.5 (see supplementary information Fig. S5). Nevertheless, this value of ΔE is still one order of magnitude larger than that obtained for non-resonant modulation^21^, where the TP mode is far detuned from the exciton polariton. Therefore, utilizing this technique to create a hybrid state of TP-microcavity exciton polariton will help to produce lateral potential channels for the polaritons. The importance of such channels for the realization of polariton based devices such as exciton-polariton transistors, switches, logic gates, and polariton integrated circuits are proposed in literature^35^,^36^. These confining channels can also be created by etching the planar MC. However, it requires much more demanding processing techniques^12^. In this context the hybrid metal-MC approach would be an interesting alternative choice.

## Discussion

We have experimentally demonstrated the existence of a hybrid state of the TP-microcavity exciton polariton by depositing a thin Ag film on top of a ZnSe-based monolithic microcavity. When the QW emission is very far detuned from the cavity mode the microreflectivity measurements show the formation of the TP and the cavity mode on the low energy side of the photonic stopband. The spectral positions of both modes exhibit a nonuniform shift to higher energies when the thickness of the top DBR layer is reduced. Further, they show an anticrossing with a splitting energy of about 44 meV. The formation of a hybrid state of the TP-microcavity exciton polariton is manifested by the observation of four resonances in the microreflectivity spectrum of the Ag covered MC sample at 4 K. They show three anticrossings when the bare TP mode is tuned. The experimental findings agree with the calculated results based on the transfer matrix method as well as on the coupled-oscillators model. A confinement for the lower polaritons being as large as about 13 meV as discussed previously is obtained when the TP is resonantly coupled with the exciton polariton. We anticipate that such a concept for the lateral confinement of the lower polariton eigenstate may be important for manipulating, shaping, and directing the flow of polaritons. Additionally, a possibility for electrical tuning could be established.

## Methods

The investigated sample was grown by molecular beam epitaxy (MBE) in an EPI 930 twin chamber system. The high-index layer of the DBR is constituted of Zn_0.72_Mg_0.28_S_0.29_Se_0.71_ with a thickness of 44 nm while the low-index layer consists of a superlattice of MgS and ZnCd_0.79_Se_0.21_ with a total thickness of 50 nm. A 1*λ* cavity was used in order to achieve a smaller mode volume which is advantageous for obtaining a larger Rabi splitting energy^11^. For the cavity, a high index layer of Zn_0.81_Mg_0.19_S_0.20_Se_0.80_ was employed. In a 1*λ* cavity, one antinode is formed at the center of the cavity^37^. Three ZnSe QWs with a thickness of 8 nm each were placed at the antinode of the electric field to achieve maximum interaction with the field. For the top layer of the upper DBR, the quaternary high-index material is used once again, which is an essential condition of the structure to support TPs^22^. The refractive index contrast between the high- and low-index material of the DBR is 0.41 at E = 2.792 eV. The eigenenergy of the TP resonance can be altered by varying either the thickness of the top layer of the DBR or by a metal layer^22^. However, increasing the metal layer thickness would diminish the transmission of the TP resonance^24^ (See Fig. S4). Hence changing the thickness of top layer of the DBR would be advantageous in order to vary the TP eigenenergy. The upper layer thickness of the top DBR of the MC sample was reduced by CAIBE. The first DBR pair of the top DBR was completely removed, hence, the number of top layer pairs has been reduced to 10. A thickness gradient for the top DBR layer was realized by further etching with a shadow mask. The thickness gradient was estimated by SEM, profilometer and by the calculation based on the transfer matrix method (software CAMFR^38^). Finally, a 40 nm thick Ag layer was deposited by electron-beam physical vapor deposition on top of the sample surface. Microreflectivity measurements with a white light source (spot diameter ~3*μ*m) were performed on different areas of the sample with and without Ag at various temperatures. A fiber coupled spectrometer with a SR = 11 meV for the overview of the reflectivity spectra and a high resolution spectrometer SR = 0.1 meV for the spectral region of interest were used. The experimental findings are compared to calculations using the transfer matrix method and the coupled-oscillator model. The parameters for the simulations like the layer thicknesses are evaluated by X-ray diffraction (XRD) and the semiconductor refractive index dispersions can be found in ref. 39. In the calculations, the Drude model was utilized to describe the refractive index of the Ag layer^40^.

## Additional Information

**How to cite this article**: Rahman, SK. S. *et al*. Observation of a hybrid state of Tamm plasmons and microcavity exciton polaritons. *Sci. Rep*. **6**, 34392; doi: 10.1038/srep34392 (2016).

## Supplementary Material

Supplementary Information
