# Supplementary material for: Observation of a hybrid state of Tamm plasmons and microcavity exciton polaritons
Source: Sci Rep. 2016 Oct 4;6:34392. doi: 10.1038/srep34392 (PMC5048173; doi:10.1038/srep34392)
Supplement: Supplementary Information [file srep34392-s1.pdf]

Supplementary information

Observation of a hybrid state of Tamm plasmons and microcavity exciton polaritons

SK. Shaid-Ur Rahman[1*], Thorsten Klein[2,3], Sebastian Klembt[2,4], Jürgen Gutowski[1], Detlef Hommel[2,5], and Kathrin Sebald[1]

[1]Semiconductor Optics, Institute of Solid State Physics, University of Bremen, Bremen, 28334, Germany

[2]Semiconductor Epitaxy, Institute of Solid State Physics, University of Bremen, Bremen, 28334, Germany

[3]Present address: BIAS, Bremer Institut f ür angewandte Strahltechnik, Klagenfurter Str. 2, 28359 Bremen, Germany

[4]Present address: Technische Physik, Universität Würzburg, Am Hubland, D-97074 Würzburg, Germany

[5]Present address: Institute of Experimental Physics, University of Wroclaw and Wroclaw Research Center EIT+, 50204 Wroclaw, Poland.

*rahman@ifp.uni-bremen.de

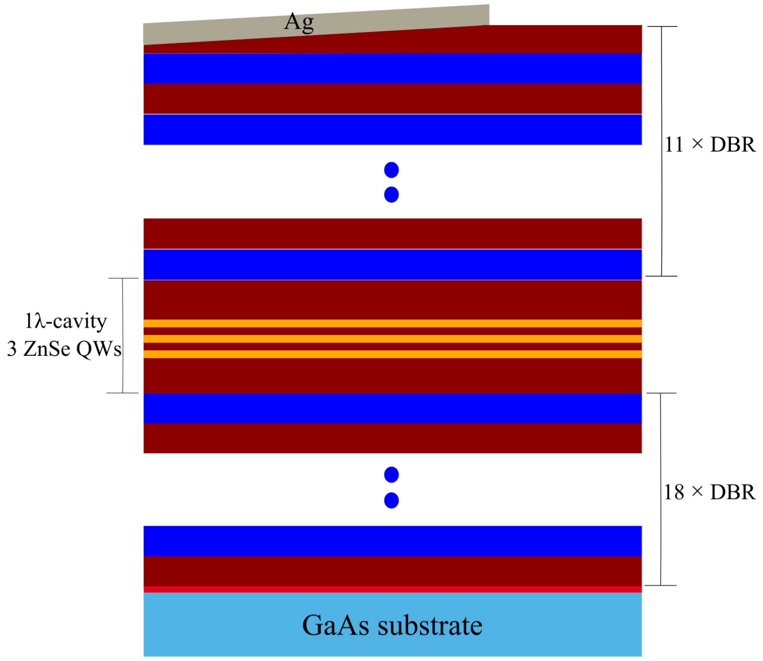

Supplementary Figure S1. Schematic of the MC structure with a 40 nm Ag layer. A thickness gradient is created in the upper layer of the 11-fold top DBR.

Here we present the influence of the number of top DBR pairs on the splitting energy between the Tamm plasmon and the cavity mode.

Fig. S1 shows the investigated sample design of the hybrid metal-MC structure. A 40 nm Ag layer is deposited onto the 11-fold top DBR. In order to vary the TP eigenenergy a thickness gradient of the top layer of the DBR is created by chemically assisted ion beam etching. Fig. S2 (a) represents the microreflectivity spectrum of the uncovered MC structure at room temperature. As expected, one cavity resonance can be identified at the spectral position of 2.792 eV. Two resonances are observed when a 40 nm Ag layer is deposited on the sample (shown in Fig. S2 (b)). The origin of the second resonance is due to the formation of the TP mode at the interface between the metal and DBR layer. Both resonances shift to higher energies when the top DBR layer thickness is reduced (Fig. S2 (c) and (d)). The dependency of the spectral position of these resonances on the top layer thickness is shown in Fig. S2 (e) in comparison to the calculated spectral positions of the resonances based on the transfer

matrix method. An anticrossing is observed between the modes in excellent agreement with calculations. The splitting energy between the modes is in the order of 34 meV which is deduced from the measurement when the TP and cavity modes are in resonance. The splitting energy for metal covered MC with a 10-fold top DBR can be determined to amount to 44 meV (shown in the main manuscript). Hence, indicates that the splitting energy is reduced with increasing the number of top DBR pairs. This observation can be explained by the longer penetration depth of the photons with increasing number of DBR pairs resulting in a reduced coupling strength between the cavity and the TP resonance.

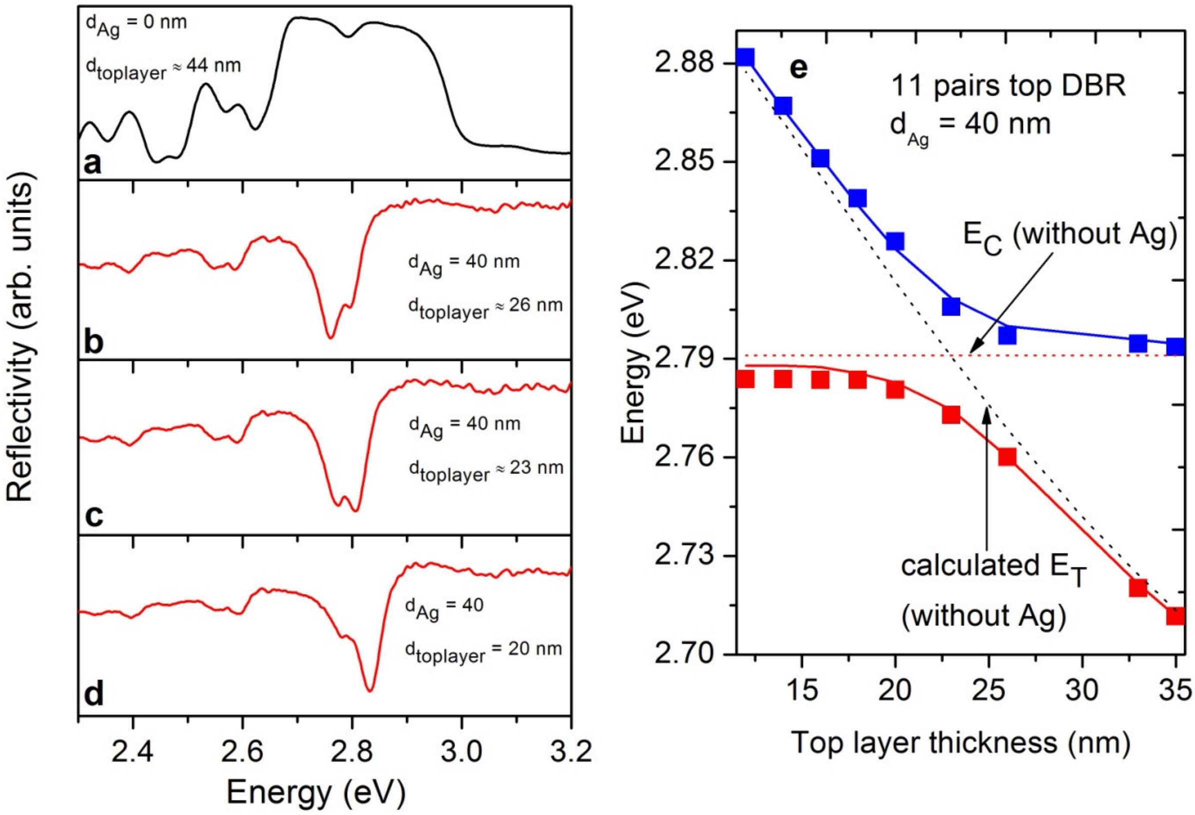

Supplementary Figure S2. Measured microreflectivity spectra of the MC sample (Fig. S1) at RT (a) without metal, (b)-(d) with a 40 nm Ag layer and different top layer thicknesses. (e) Measured (dots) and calculated spectral positions (solid lines) of the resonances of the Ag covered MC sample as function of the top layer thickness. The calculated bare TP and the bare cavity modes are shown as dotted black and red line, respectively.

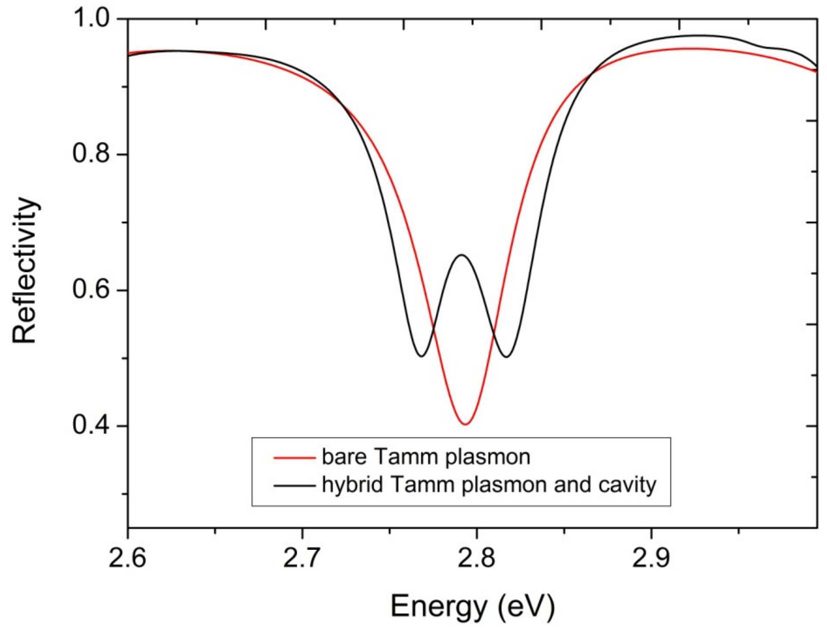

Supplementary Figure S3. Simulated microreflectivity spectrum (region of interest) of the bare Tamm plasmon (red), and the hybrid Tamm plasmon-cavity system (black lines) respectively.

Here we discuss the difference between the bare Tamm plasmon and the hybrid Tamm plasmon-cavity system.

In the calculation it was assumed that the TP structure consists of a DBR and a 40 nm Ag layer, whereas the hybrid structure consists of top DBR, cavity, and bottom DBR with a 40 nm Ag layer on top (as shown in Fig. 1(a)). Fig. S3 shows mode formation of the bare TP structure (red) and the hybrid TP-cavity structure (black line). The upper layer of the top DBR was adjusted so that the TP mode is in resonance with the cavity mode. Two nearly symmetric modes can be observed which further verifies our experimental observation (Fig 2(c) and S2 (c)). One of these modes Q factor is increased by a factor of 2 with respect to the bare TP mode due to the reduction of the metal absorption losses in the hybrid structure. Such

an enhancement of the Q factor makes this hybrid system more attractive compared to bare TP system.

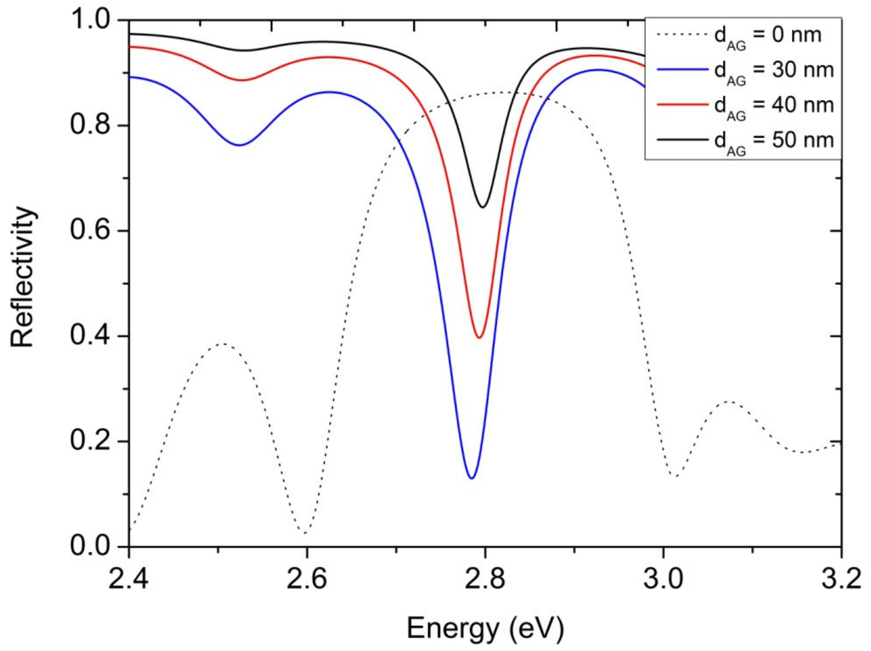

Supplementary Figure S4. Simulated microreflectivity spectra of the bare Tamm plasmon structure for different Ag layer thicknesses.

Optimized Ag layer thickness:

The TP eigenenergy can be varied by changing either the top layer or the metal layer thickness. However, the metal absorption losses increase by increasing the metal layer thickness. Therefore, altering the DBR top layer thickness would be favorable in order to tune the eigenenergy without reducing the quality of the structure. Fig. S4 shows the calculated reflectivity for different Ag layer thicknesses. The TP mode shifts to higher energies by increasing Ag thickness accompanied by an increase of the Q factor. However, the transmission of the TP mode diminishes by the enhancement of the Ag thickness. Hence, we need an optimum metal layer thickness in order to have sufficient transmission and relatively

high Q factor. Ag thickness of 40 nm is an optimum trade-off between the Q factor and the mode transmission.

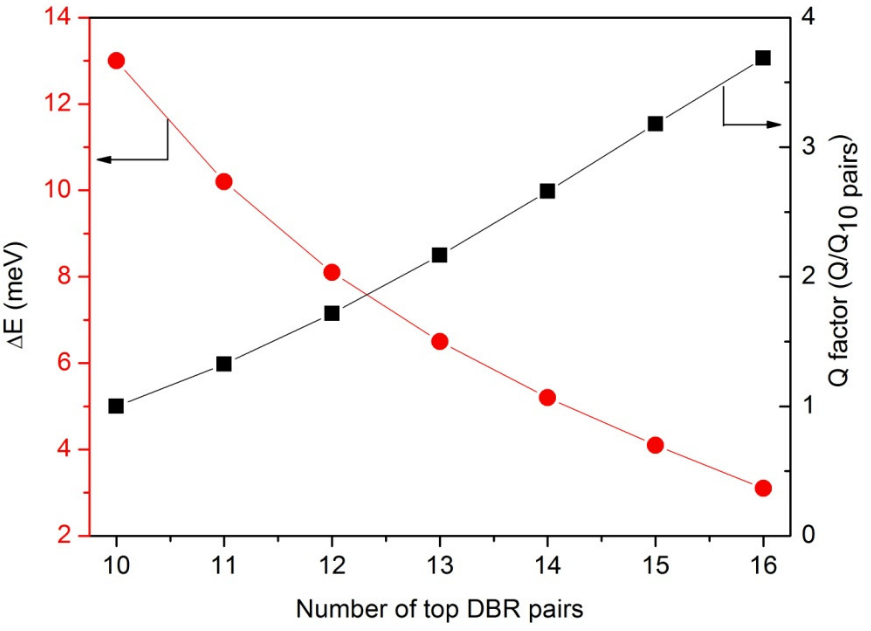

Supplementary Figure S5. The calculated lower polariton energy shift ΔE at 4K (red) and the cavity Q factor (normalized to the Q factor of the MC with a 10-fold top DBR, black-symbols) as a function of the number of top DBR pairs. Red and black lines are shown for the guidance of the eye.

Influence of the variation of the number of top DBR pairs on the cavity Q factor as well as on the lower polariton (LP) energy shift ΔE:

The LP energy shift ΔE with respect to the LP position of the metal free MC can be varied by changing the number of top DBR pairs as shown in Fig. S5 (red-symbols). In the calculation the structure of the MC was assumed as shown in Fig. 1 (a) and the thickness of the upper layer of the top DBR layer was adjusted so that the heavy-hole exciton (X_{hh}), cavity, and TP

mode are in resonance (E_{hh} = E_C = E_T). From the calculation we can observed that the confinement potential ΔE reduces by increasing the number of DBR pairs. The reason for the reduction of ΔE is the same as we have discussed for the bare interaction between the TP and cavity modes (first part of the supplementary information). However, the cavity Q factor enhances by increasing the number of top DBR pairs as expected (Fig S5 (black-symbols)). Nevertheless, the value of ΔE is still in the meV range when the Q factor is enhanced by a factor of about 3.5. Therefore, this hybrid approach is also suitable for the high Q MC system.
